# XPG: a multitasking genome caretaker

**DOI:** 10.1007/s00018-022-04194-5

**Published:** 2022-03-01

**Authors:** Alba Muniesa-Vargas, Arjan F. Theil, Cristina Ribeiro-Silva, Wim Vermeulen, Hannes Lans

**Affiliations:** Department of Molecular Genetics, Erasmus MC Cancer Institute, Oncode Institute, Erasmus University Medical Center, Rotterdam, The Netherlands

**Keywords:** DNA damage response, XPG/ERCC5, Structure, NER, Xeroderma pigmentosum–Cockayne syndrome

## Abstract

The XPG/ERCC5 endonuclease was originally identified as the causative gene for *Xeroderma Pigmentosum* complementation group G. Ever since its discovery, in depth biochemical, structural and cell biological studies have provided detailed mechanistic insight into its function in excising DNA damage in nucleotide excision repair, together with the ERCC1–XPF endonuclease. In recent years, it has become evident that XPG has additional important roles in genome maintenance that are independent of its function in NER, as XPG has been implicated in protecting replication forks by promoting homologous recombination as well as in resolving R-loops. Here, we provide an overview of the multitasking of XPG in genome maintenance, by describing in detail how its activity in NER is regulated and the evidence that points to important functions outside of NER. Furthermore, we present the various disease phenotypes associated with inherited XPG deficiency and discuss current ideas on how XPG deficiency leads to these different types of disease.

**Supplementary Information:**

The online version contains supplementary material available at 10.1007/s00018-022-04194-5.

## Introduction

DNA lesions are an unavoidable fact of life and it is estimated that, daily, each of our cells is confronted with approximately 10^4^–10^5^ new DNA lesions [1, 2]. These lesions can interfere with essential genome processes, such as transcription and replication, and thus have immediate and long-term consequences. Cells, therefore, utilize a range of specialized DNA repair mechanisms, signaling pathways, tolerance processes and cell cycle checkpoints, collectively called the DNA damage response (DDR), to cope with DNA lesions and maintain proper function of the genome [1, 3, 4]. Genetic diseases, neurological degeneration, premature aging and increased cancer susceptibility are severe fallouts of inherited DDR defects that illustrate the human’s health reliance on an operational DDR. XPG, also called ERCC5, is a major DDR endonuclease, whose deficiency results in severe developmental defects, progeria and cancer. It is mainly known for its role in excising DNA damage in nucleotide excision repair (NER), but in recent years, it has been found to function in other genome maintenance mechanisms as well. In this review, we provide a detailed overview of XPG’s function and activity in NER, highlighting recent new insights, discuss the evidence suggesting that it has important functions beyond NER and describe the pleiotropic phenotypic consequences of inherited XPG deficiency.

## Nucleotide excision repair

**Fig. 1:**
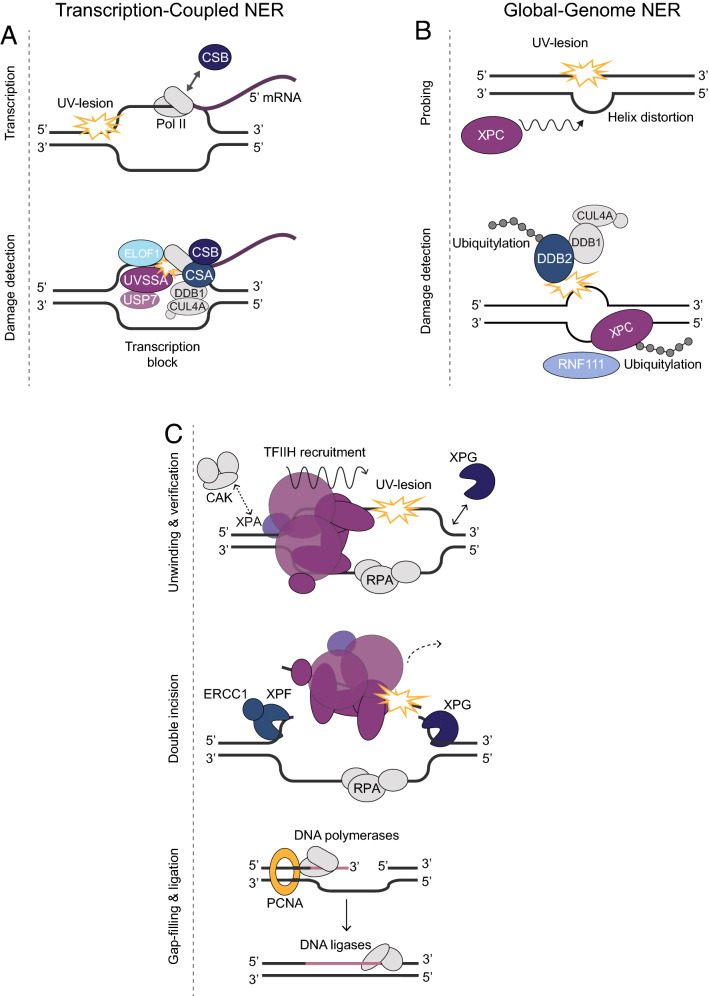
Nucleotide excision repair mechanism. **A** Transcription-coupled NER. Pol II stalling at UV lesions recruits CSB, whose prolonged binding to Pol II triggers CSA recruitment, which is part of the larger CRL4^CSA^ complex that also comprises DDB1, CUL4A and RBX1. CRL4^CSA^ interacts with ELOF1 and ubiquitylates CSB and Pol II to target these for proteasomal degradation. Next, UVSSA and USP7 are recruited, which, respectively, recruit TFIIH and de-ubiquitylate and stabilize CSB. **B** Global genome NER. DDB2, as part of the CRL4^DDB2^ complex, binds to UV lesions and facilitates their efficient recognition and stable binding by XPC, by means of auto-ubiquitylation and XPC ubiquitylation. Stable binding of XPC leads to TFIIH recruitment, followed by RNF111-mediated ubiquitylation and dissociation of XPC. **C** Core NER reaction. Stable association of XPC or UVSSA to lesions recruits TFIIH, which unwinds the DNA with its helicase activity to verify the damage. XPA displaces the TFIIH CAK subcomplex and stimulates its helicase activity. RPA binds the undamaged strand and together with XPA positions the ERCC1–XPF and XPG endonucleases 5ʹ and 3ʹ to the lesion, respectively. XPF 5ʹ incision is followed by XPG 3ʹ incision after which PCNA and DNA polymerases, together with other re-synthesis factors, are recruited to fill the gap

NER is unique in its ability to repair a wide range of lesions that arise from diverse and different genotoxic insults because, in contrast to most other DNA repair pathways, it detects the structural consequences of DNA damage, i.e., helix destabilization, instead of the DNA lesion itself [5]. These helix-distorting lesions include the UV-induced cyclobutane pyrimidine dimers (CPDs) and pyrimidine-pyrimidone (6–4) photoproducts (6–4PPs), ROS-induced cyclopurines and chemotherapy drug-induced (e.g., cisplatin) intrastrand crosslinks. More than 30 proteins are involved in the intricate network of NER, and cooperate to perform four essential steps: (1) damage detection; (2) damage verification; (3) excision of a single-stranded damage-containing DNA segment; and (4) DNA synthesis and ligation to restore and seal the gap [6, 7] (Fig. 1). Depending on where in the genome lesions occur, two different damage detection sub-pathways can initiate NER. Transcription-coupled repair (TC-NER) detects lesions in the transcribed strand of active genes, whereas global genome repair (GG-NER) detects lesions anywhere in the genome.

The great majority of helix-destabilizing DNA lesions are detected by GG-NER, which examines the entire genome, coding and non-coding, for DNA damage-induced helical distortions [6, 7]. The XPC protein, as part of the heterotrimeric XPC–CETN2–RAD23B complex is capable of detecting a broad range of structurally unrelated lesions by employing an indirect damage recognition mode with which it probes for DNA helix destabilization [5, 8, 9]. XPC requires the auxiliary function of the CRL4^DDB2^ E3 ubiquitin ligase complex, comprising DDB1, DDB2, CUL4A and RBX1, to efficiently recognize some types of lesions, such as the UV-induced CPDs [10, 11]. CRL4^DDB2^ stimulates XPC recruitment by binding and flipping out damaged bases, making them more suitable substrates for XPC, and by ubiquitylating both DDB2 and XPC, promoting the DNA damage handover [10–14]. To counteract the cytotoxic effects of lesions that block RNA Polymerase II (Pol II) forward translocation during transcription elongation, TC-NER is activated with the recruitment of CSB and CSA proteins [15, 16]. CSB transiently interacts with Pol II, but binds more stably when it cannot push Pol II forward due to a transcription-blocking lesion [17]. This leads to recruitment of CSA, which is part of the larger E3 ubiquitin ligase CRL4^CSA^ and directs the ubiquitylation and proteasomal degradation of CSB and, after interacting with ELOF1, of Pol II [18–22]. CSB is, however, stabilized by subsequent recruitment of the UVSSA protein together with the de-ubiquitylation enzyme USP7 [23–25].

Stable DNA damage association of XPC, in GG-NER, or UVSSA, in TC-NER, leads to the next step of damage verification by the NER machinery through recruitment of TFIIH, which directly interacts with either XPC (via GG-NER) or UVSSA (via TC-NER) [26–28]. TFIIH is a 10-subunit multifunctional complex that opens the DNA helix in both NER [29] and transcription initiation by Pol II [30]. Its XPD helicase subunit verifies the presence of genuine NER substrates by unwinding the DNA in 5ʹ–3ʹ direction while scanning for helicase-blocking lesions [29, 31]. This helicase activity and damage verification is stimulated by the association of the DNA damage binding protein XPA [31–33], which also, together with the single-stranded DNA-binding factor RPA, orients the two structure-specific endonucleases ERCC1–XPF and XPG on the damaged strand [34–36]. The presence of XPG enables the first incision, 5ʹ to the lesion, by ERCC1–XPF, and the second incision, 3ʹ to the lesion and leading to damage excision, is then finalized by XPG itself [36]. The resulting 22–30 nucleotide DNA gap is filled by novel DNA synthesis involving the activity of replication proteins RFC, PCNA and either DNA polymerase *δ* (non-replicating cells), *ε* (mainly in replicating cells) or *κ* (non-replicating cells) and either DNA ligase I or III to seal the gap [37–39].

## XPG activity in nucleotide excision repair

**Fig. 2:**
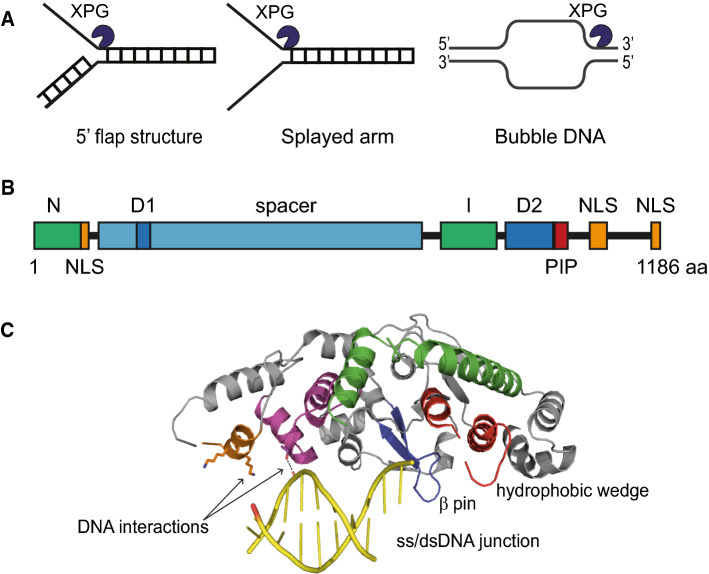
XPG structure and DNA substrate binding. **A** DNA substrates of XPG. XPG binds to and incises 5ʹ flap structures, 5ʹ single-stranded tails of splayed arms, and the 3ʹ junction of DNA bubbles. **B** XPG schematic structure. XPG contains two nuclease domains N and I, separated by a unique spacer region, a PCNA-interacting Protein (PIP) box and three Nuclear Localization Signal (NLS) regions. The D1 and D2 boxes are highly conserved among eukaryotes. **C** XPG interactions with DNA. Crystal structure of the catalytic core of XPG in complex with a splayed-arm DNA substrate. XPG interacts with dsDNA via a helix-2-turn-helix (H2TH) module (shown in purple) and an adjacent α-helix (shown in orange). Furthermore, a hydrophobic wedge and β-pin interact with the ss/dsDNA junction. Image depicts structure PDB 6TUW (complex 1) from [76] and was generated using PyMol

XPG is a member of the XPG/RAD2 family of structure-specific nucleases, which in mammals also includes FEN1, GEN1 and EXO1 and which all have important genome maintenance functions [40]. FEN1 participates in DNA replication by cleaving 5ʹ single and double flap structures [41]. GEN1 functions in resolution of double holiday junctions during various types of homologous recombination [42]. EXO1 resects DNA in multiple genome maintenance mechanisms, including mismatch repair, double-strand break repair and NER [43]. XPG is an endonuclease that was found to cut 5ʹ flap structures, 5ʹ single-stranded tails of splayed-arm structures and to incise bubble DNA at the 3ʹ junction in vitro [44, 45] (Fig. 2a). This activity implies that during NER, XPG incises DNA at the 3ʹ site of the lesion. Indeed, incision of XPG during NER requires a bubble substrate [46], which is generated by the helicase activity of TFIIH [47, 48]. As XPG interacts with DNA at ss/dsDNA junctions [49], XPG is likely only stably bound to damaged DNA in vivo once this bubble is created, i.e., simultaneously with or after TFIIH recruitment and activity. Indeed, its recruitment and stable binding to DNA damage in living cells was shown to depend on functional TFIIH and to be temperature sensitive, in line with a role for TFIIH helicase activity to unwind DNA [35]. The TFIIH-dependent recruitment of XPG furthermore correlates with the strong observed interaction between XPG and TFIIH, even in the absence of DNA damage, suggesting that at least a fraction of XPG is always bound to TFIIH and stabilizes it [50, 51]. Besides, XPG has been reported to interact directly with RPA [52], which also stimulates its incision activity in vitro [53]. As RPA binds the undamaged ssDNA opposite of the DNA lesion after TFIIH has unwound the DNA, this interaction is probably important for stable association and/or positioning of XPG [34].

XPG is a 1186-amino acid protein consisting of two conserved nuclease domains, i.e., the N and I region, which it shares with other nucleases but which are separated by a spacer region that is unique to XPG [45, 54] (Fig. 2b). In addition, two regions were identified that are strongly conserved among higher eukaryotes, referred to as the D1 and D2 boxes [55], of which the first overlaps with a predicted ubiquitin binding motif (UBM) [56]. Also, XPG contains three nuclear localization sequences and a PIP-box motif for interaction with PCNA [57, 58]. Multiple subunits of TFIIH, including XPB, XPD, p52 and in particular also p62, via its Pleckstrin homology (PH) domain, were found to interact with multiple sites within XPG [32, 59, 60]. Structural and genetic studies of the yeast XPG homolog Rad2 identified two acidic segments within the Rad2 spacer region (Rad2_642–690_ and Rad2_359–383_) that have high affinity for the PH domain of Tfb1, the yeast homolog of p62, and functionally stimulate NER [61, 62]. The PH motifs of yeast Tfb1 and human p62 are very similar [63], suggesting functional conservation even though the sequence of the spacer region in XPG is not strongly conserved. Indeed, chemical crosslinking of purified XPG, in complex with TFIIH, XPA and a DNA substrate, suggests a direct interaction of the spacer region with p62 [32]. Moreover, the disordered C-terminus of XPG appears to mostly interact with XPB and p52, while the N-terminal part of the spacer region mostly interacts with XPD. Interestingly, deletions specifically of the N-terminal part of the spacer region were found to disrupt interactions with TFIIH and impair XPG endonuclease activity, causing an increase in UV sensitivity in cells [64, 65].

In GG-NER, upon damage detection, XPC recruits TFIIH to sites of damage via interactions with its XPB and p62 subunits, as indicated by binding experiments with purified proteins [27, 66]. Because XPB and p62 also appear to be involved in XPG recruitment [59, 60] and because in in vitro reconstituted NER assays XPG binding to DNA damage coincides with XPC dissociation [67, 68], it is thought that XPG exchanges with XPC upon recruitment to DNA damage. In fact, XPC and its yeast ortholog Rad4 were shown to contain similar acidic binding motifs as XPG/Rad2 with which they interact with the PH domain of p62/Tfb1 and compete for binding with XPG/Rad2 [69, 70]. The idea that XPC and XPG exchange is further supported by live cell imaging studies showing that inefficient XPC dissociation, due to inhibition of its RNF111-mediated ubiquitylation, impairs the stable integration of XPG into active NER pre-incision complexes [71]. It is, however, currently unclear how this exchange of XPC and XPG binding to TFIIH can be reconciled with the idea that XPG is already bound to TFIIH in the absence of DNA damage [50, 51]. Possibly, this involves structural changes and/or dimerization of XPG, as has been recently suggested to occur [72], which would allow XPG to bind in an alternate fashion and more tightly to TFIIH upon DNA damage recruitment and would position XPG for DNA incision.

It is often assumed that XPG is similarly recruited by its interaction with TFIIH in TC-NER, but this has not been formally shown. After initiation of TC-NER by binding of CSB to lesion-stalled Pol II, CSA is recruited, which, together with ELOF1, promotes recruitment of the UVSSA protein [15, 16, 21, 28]. Subsequently, TFIIH is recruited by interaction with UVSSA, which has a similar acidic motif as XPC and XPG and binds to TFIIH through the PH domain of p62 [26, 28]. Therefore, it is conceivable that, in TC-NER, XPG exchanges binding to TFIIH with UVSSA as it does with XPC in GG-NER. It is, however, unclear whether TFIIH has a similar role in opening DNA in TC-NER as in GG-NER, to generate the proper substrate for XPG incision, as within the transcription bubble of lesion-stalled Pol II the DNA is already unwound [15]. Therefore, it is possible that XPG recruitment and its stable association to TC-NER complexes involve additional or different mechanisms. In support of this, it was found that in vitro XPG can interact directly with stalled Pol II and CSB in a transcription-sized DNA bubble [59, 73]. Also, TFIIH was found to interact with the RPB6 subunit of Pol II directly via the PH domain of p62 [74], implying that recruitment and/or stable association of XPG with TC-NER complexes additionally involves direct interactions of XPG and TFIIH with stalled Pol II. Interestingly, incision of the transcription bubble by XPG was inhibited by stalled Pol II in the absence of TFIIH [73], which suggests that in TC-NER TFIIH activity may be required to remodel lesion-bound Pol II to permit DNA incision.

## XPG structure, DNA-binding and dimerization

Several structure-based studies have provided important insights into XPG’s DNA binding, substrate specificity and incision. DNA-binding and footprinting assays with purified XPG showed that XPG mainly binds to the dsDNA part of a splayed-arm structure and cuts 1 nt into the DNA duplex, preferably if a 5ʹ ssDNA overhang is present [49]. Crystallization of the catalytic core domain of Rad2 and XPG in complex with a DNA substrate furthermore revealed that Rad2/XPG interacts via several structural motifs with dsDNA and the ssDNA/dsDNA junction that mimic the NER DNA bubble structure [75, 76]. The key interactions with DNA are mediated by a helix-2-turn-helix (H2TH) module of XPG with a flanking α-helix that binds both strands of the dsDNA (Fig. 2C). The other key contact is with the ss/dsDNA junction and is mediated by a ‘hydrophobic wedge’ and β-pin that forms a protrusion. Interestingly, the human XPG structure shows the existence of a helical arch pore suggesting the possibility of threading of the 5ʹ DNA flap before incision [76], but this needs further investigation. Although not evident from these crystal structures, it is interesting to note that there are indications that XPG acts as a dimer. Whether XPG acts in NER as mono- or multimer has been for long a topic of debate. XPG appears to diffuse freely through the nucleus without being part of a larger complex [35] and when purified exists as monomer in high salt conditions, but co-immunoprecipitation experiments of XPG with itself from insect or human cells suggest that XPG can form dimers [72, 77]. Also, purification of XPG through size-exclusion chromatography shows a peak of which the molecular mass is in accordance with a dimer. Mutation of residues in a putative dimer interface of XPG led to protein destabilization and reduced incision activity of a bubble substrate [72]. Intriguingly, dimerization could imply that XPG interacts with dsDNA on both sides of a NER DNA bubble structure, which could be functionally relevant to XPF-mediated incision, as discussed below.

## Non-catalytic functions and dissociation of XPG from DNA damage

XPG does not only have a catalytic but also a structural role in NER. XPG stably associates with TFIIH and stabilizes the complex [51] and, in vitro, stimulates XPD helicase activity and DNA opening by TFIIH [32]. Cryo-EM and chemical crosslinking studies of TFIIH, XPA and XPG bound to a DNA substrate suggest that the XPB translocase and XPD helicase activities are inhibited by interaction of XPD with the CAK (cyclin-dependent kinase activating kinase) TFIIH subcomplex formed by MNAT1, CDK7, and Cyclin H. The CAK subcomplex mediates important transcription functions of TFIIH, such as, e.g., phosphorylation of Pol II, but is dispensable for NER [30]. Binding of XPA stabilizes a TFIIH conformation that cannot bind CAK, in line with the observation that XPA stimulates dissociation of this subcomplex from TFIIH during NER [33], which would, thus, relieve this inhibition. Interestingly, the N-terminus of XPG also interacts with XPD at its binding site for the CAK subcomplex, suggesting that competition with CAK for binding to XPD is how XPG can stimulate XPD helicase activity.

Upon recruitment of TFIIH, XPA, RPA and XPG to DNA damage, damage removal is initiated by DNA incision at the 5ʹ site of the lesion by ERCC1–XPF, such that repair synthesis can also be started. This depends on the structural presence of XPG but does not require its catalytic activity [36, 78]. Only after ERCC1–XPF incision, XPG cuts at the 3ʹ site of the lesion. It is currently unclear how the mere presence of XPG at the NER bubble substrate facilitates ERCC1–XPF-mediated incision and, vice versa, how its 5ʹ incision subsequently provokes 3’ incision by XPG. It has been suggested that XPG dimerization might allow XPG to bind both 3ʹ as well as 5ʹ to the NER bubble substrate, close to XPF, thus facilitating its incision [72]. Furthermore, XPG activation and incision has been hypothesized to be induced by a conformational change in the NER incision complex or by a structural rearrangement in XPG itself, such as a posttranslational modification, due to either ERCC1–XPF-mediated 5’ incision or due to initiation of DNA repair synthesis following this 5ʹ incision [6, 36, 54].

Following dual incision, it is unclear what exactly happens to XPG. Some NER proteins that have performed their activity are actively unloaded, involving posttranslational modifications and the activity of other proteins, such as DDB2 by the segregase VCP/p97 [14, 79] and XPC by the E3 ubiquitin ligase RNF111 [71]. The dissociation of other NER proteins appears to coincide with the release of the excised damaged DNA oligomer. Excision assays in cell-free extracts and cells showed that NER-excised DNA fragments are in tight complex with TFIIH [80], but also with XPG, possibly because of its strong association to TFIIH [81]. This suggests that XPG might dissociate along with the excision product upon incision. However, it has been suggested that, besides incising DNA, XPG is involved in DNA re-synthesis by helping to recruit DNA synthesis factors, in particular PCNA, indicating that XPG initially remains bound after incision. In vitro reconstitution of dual incision of a cisplatin–DNA adduct suggested that excision coincides with the dissociation of several factors, including TFIIH and XPF, but that XPG and RPA remain bound to DNA to participate in DNA re-synthesis, likely by helping recruit RFC and PCNA [67, 82]. Recruitment of PCNA to DNA damage indeed depends on XPG [83], but so does the 5ʹ incision by XPF [36]. In the presence of nuclease-inactive XPG, ERCC1–XPF can incise DNA and post-incision NER factors, including PCNA, are recruited, pointing to the possibility that DNA repair synthesis can be initiated before 3ʹ incision by XPG takes place [36]. XPG has been implicated in recruiting PCNA through a direct interaction with a PIP-box motif in its C-terminus [57]. PCNA furthermore interacts with XPA [84], which might regulate its recruitment, and RFC, which is needed to properly load the PCNA clamp around DNA [85]. Thus, it was postulated that XPG recruits PCNA before incising the damage at the 3ʹ site after which PCNA is correctly loaded by RFC after 5ʹ incision and XPF dissociation [86]. Since it was found that XPG is bound to excised DNA fragments [81], this suggests that even though XPG may initially, before or during 5ʹ DNA incision, help to recruit PCNA, it rapidly dissociates together with the excised DNA fragment once the dual incision takes place, handing over PCNA to RFC and possibly XPA. Alternatively, it was proposed that upon incision and recruitment of PCNA, XPG is ubiquitylated by the E3 ubiquitin ligase complex CRL4^Cdt2^ and subsequently degraded, to make room for subsequent DNA synthesis factors [87]. However, even though ubiquitylation of XPG after UV has been reported by others as well [88, 89], its precise function and whether this indeed promotes XPG removal, requires further confirmation.

## XPG functions beyond NER

### Transcription and base excision repair

**Fig. 3:**
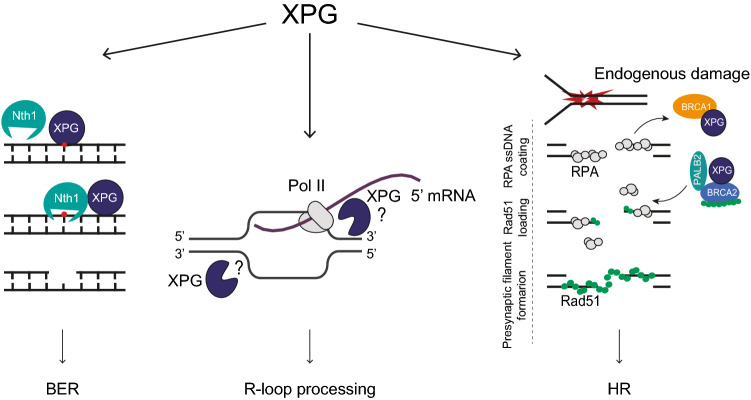
XPG functions in genome maintenance pathways other than NER. **A** Repair of oxidative damage. Initial binding of XPG to an oxidized base stimulates Nth1 DNA-binding activity, followed by removal of the oxidized base and repair by BER. **B** R-loop processing. R-loops arise when during Pol II transcription the nascent mRNA hybridizes with one of the DNA strands. One of the 3ʹ prime junctions of the resulting bubble structure is recognized and processed by XPG endonuclease activity. **C** Homologous Recombination. HR of DSBs generated by endogenous DNA damage is initiated by DNA end resection followed by RPA coating of the resulting ssDNA. XPG interacts with and facilitates the loading of the BRCA2–PALB2–RAD51 complex to DSB sites. RAD51 replaces RPA and promotes the presynaptic filament to initiate HR. Of note, closed XPG circles represent putative non-catalytic activity and open XPG circles represent catalytic activity of XPG

Besides being involved in NER, XPG has been implicated in other DNA transacting and maintenance mechanisms (Fig. 3). Together with other NER proteins, XPG has been implicated in regulating transcription, both by stabilizing TFIIH [51] as well as by promoting demethylation and, through the generation of DNA breaks, recruitment of the chromatin organizer CTCF to gene promoters [90, 91]. Also in yeast, Rad2/XPG was implicated in regulating transcription through an interaction with Pol II and Mediator [92, 93]. Furthermore, XPG is implicated in the removal of oxidative DNA damage by base excision repair (BER). This pathway resembles NER in that it mediates excision of a damaged base, but both the detection of oxidative base lesions, by various glycosylases, as well as the removal of the lesion and gap filling are performed by a different set of proteins. Still, emerging evidence suggests that substantial interplay between the NER and BER machineries exists [94] and multiple NER proteins have been implicated in stimulating BER activity, even though the precise involved mechanisms are not yet understood [95–99]. Cells deficient in several NER proteins, including XPG, show reduced repair of and/or are hypersensitive to oxidative DNA damage [100], suggesting a function in the removal of oxidative DNA lesions. Interestingly, in an in vitro reconstituted BER system, XPG was found to stimulate the DNA-binding activity of the DNA glycosylase NTH1, which detects and removes oxidized bases, independently of its endonuclease activity [101, 102]. Interaction between XPG and NTH1 was furthermore confirmed in cells [103]. These data suggest a possible role for XPG in the removal of oxidative lesions that is independent from NER, but further studies, especially cellular or in vivo experiments*,* are needed to support this notion.

### Resolving R-loops

One of the major activities of XPG besides NER appears to be in resolving R-loops. These are three-stranded RNA–DNA hybrid-containing structures that originate during transcription when the nascent RNA molecule from the transcription machinery hybridizes with the DNA template strand. Physiological R-loops are commonly found in many organisms and are implicated in transcription regulation. However, pathological R-loops may arise in an unscheduled fashion, for instance due to transcription blockage or defects in RNA processing, and by interference of DNA replication with transcription [104]. Cells have developed multiple specialized mechanisms to cope with these R-loops, including RNA degradation by ribonucleases and RNA–DNA hybrid unwinding by helicases. When these mechanisms fail, RNA–DNA hybrid structures can lead to DNA damage and genomic instability, although they are thought to play a faciliatory role in DDR as well [105, 106]. Interestingly, since R-loops form bubble structures that resemble substrates for ERCC1–XPF and XPG, both NER endonucleases are considered as candidates to process R-loops. Indeed, ERCC1–XPF and XPG can cleave R-loops in vitro [107]. Furthermore, it was observed in cells that the depletion of the R-loop helicase AQR resulted in more R-loop formation and fewer DNA breaks if XPF or XPG are deficient [108]. Because of these and similar findings with other TC-NER factors, it was proposed that TC-NER processes R-loops associated with paused transcription into double-strand breaks (DSBs) [108, 109]. Interestingly, in line with this idea, it was found that the HLTV-1 viral oncoprotein Tax promotes cellular senescence by inducing an increase in R-loops that results in excessive DSB formation by XPF and XPG [110]. Therefore, HTLV-1-induced adult T-cell leukemias avoid senescence by selection for cells that have lost the activity of XPG or other TC-NER proteins.

XPG was also found to be recruited to R-loops generated at DSBs in transcriptionally active regions, in a Rad52-dependent manner, to help resolve R-loops and stimulate repair by transcription-associated homologous recombination [111]. Additional evidence that XPG processes R-loops comes from the observations that XPF and XPG form a complex together with the splicing factor XAB2, which is independent of NER, that is targeted to R-loops [112]; that XPG co-localizes with R-loops [113]; and that there is reduced DSB formation upon XPG depletion in cells with increased R-loops, due to camptothecin-induced transcription blockage [114] or due to replication-fork stalling in Werner protein deficient cells [115]**.** Together, these results strongly suggest that XPG is an important factor for cells to deal with R-loops, but it remains to be investigated to which types of R-loops and processing mechanisms XPG is important and with which other R-loop processing factors it interplays. Contradicting results still exist and should be resolved before a clear understanding of XPG function in regulating R-loop metabolism is obtained. For instance, both XPF and XPG, as part of the whole TC-NER machinery, were implicated in resolving R-loops when other mechanisms that prevent their excessive formation are inhibited [108]. On the contrary, only XPG, but not XPF, was found to be recruited to and help resolve R-loops at DSBs in transcriptional active regions [111]. This may suggest that XPG can mediate R-loop processing independently from (TC-)NER, but it is unclear how XPG is recruited and, especially, positioned and activated to cleave R-loops. Given the tight regulation of XPG (and XPF) function in NER, by multiple protein–protein interactions, it seems reasonable to assume that similar mechanisms exist to prevent unwanted XPG-mediated incisions during R-loop metabolism. How this is accomplished is currently unknown and should be further investigated.

### Homologous recombination and replication stress

Besides processing R-loops at stalled replication forks [115], XPG may also function non-enzymatically to counteract replication stress. Purified XPG binds fork-like structures [49, 72] and in cells XPG was shown to interact and colocalize at stalled forks with the WRN helicase, which is thought to serve as fork-protection factor during replication stress [116]. Interestingly, independently of its catalytic activity, XPG was able to stimulate both the helicase and annealing activity of WRN in vitro*.* Furthermore, XPG was found to have a non-catalytic role in recovery from replication stress by promoting homologous recombination [117]. XPG interacts with multiple homologous recombination proteins, including BRCA2, PALB2 and RAD51, and promotes their loading at DSB sites. In addition, XPG interacts with BRCA1 and promotes its dissociation from chromatin [117]. In spite of these intriguing findings, much of the proposed functions of XPG in replication stress and homologous recombination still await further confirmation and should be investigated in more detail to precisely understand the structural role of XPG in maintaining genome stability of replicating cells. Also, in transcriptionally active chromatin, XPG recruitment to R-loops at DSB sites may indicate that XPG can have a double role in promoting HR. First, XPG may help resolve R-loops by its endonucleolytic activity, after which it may further promote repair by its association with HR factors. It would therefore be interesting to investigate the connection between these enzymatic and non-enzymatic functions in HR.

## XPG and human disease

The multiple tasks that XPG performs both enzymatically and structurally in genome maintenance, replication and transcription imply that it must be an essential and critical factor for cellular homeostasis and organismal development and growth. Indeed, hereditary mutations in XPG are associated with several rare human diseases characterized by a perplexing broad spectrum of symptoms including cancer predisposition, progressive neurodegeneration and developmental failure (Tables 1, 2, 3; Supplementary Table 1). Mild XPG mutations cause xeroderma pigmentosum (XP), which is characterized by photosensitivity, abnormal skin pigmentation, increased risk of cancer and sometimes, but not always, neurological diseases (Table 1). Many of these patients express XPG mutant proteins carrying a missense or frame-shift mutation near the catalytic core that disrupts or reduces XPG DNA-binding and/or nuclease activity and, thus, NER efficiency [76, 77]. XP symptoms are therefore thought to be mainly caused by reduced or defective GG-NER, in which the degree of the repair defect will correlate with the severity of symptoms. More severe mutations, which often truncate the XPG protein or, in case of missense mutations, are thought to disrupt the entire protein function, stability and interactions [72], cause additional Cockayne syndrome features, called xeroderma pigmentosum–Cockayne syndrome (XPCS) complex (Table 2), also referred to as cerebro-oculo-facio-skeletal (COFS) syndrome when very severe (Table 3) [118]. XPCS complex patients are characterized by mental retardation, bird-like faces, dwarfism and developmental delay, progeria and severe, progressive neurological abnormalities.

**Table 1:** XP patients with XPG/ERCC5 mutations

XP patient ID^a^	Allele 1		Allele 2^b^		Age	Age of death	Clinical features individual								
	AA change	Genomic mutation	AA change	Genomic mutation			Photosensitivity	Skin Cancer	Developmental delay	Mental retardation	Skeletal abnormalities	ophthalmologic abnormalities	psychomotor impairment	Cellular DNA Repair defect	References
Patient case	p.[Gly2Trp]	c.[4G>T]	p.[Gln233*]	c.[697C>T]	17 y	ND	N	N	N	Y^d^	N	Y	N	ND	[133]
XP01RJ	p.[Ala28Asp]	c.[83C>A]	p.[Trp968Cys]	c.[2904G>C]	22 y	ND	Y	N	N	N	N	N	N	Y	[134]
XP02RJ^c^	p.[Ala28Asp]	c.[83C>A]	p.[Trp968Cys]	c.[2904G>C]	17 y	ND	Y	N	N	N	N	N	N	Y	[134]
XP915	p.[Gln37*]	c.[109C>T]	p.[Pro147Leufs*23]	c.[440delC]	4 y﻿	ND	N	N	N	N	N	N	N	ND	[135]
XP918	p.[Leu65Pro]	c.[194T>C]	p.[?]	c.[?]	4 y	ND	N	N	N	N	N	N	N	ND	[135]
XP3HM	p.[Leu65Pro]	c.[194T>C]			40 y	ND	Y	Y (40 y)	N	N	ND	N	ND	Y	[136]
XP65BE	p.[Gln136*]	c.[406C>T]	p.[Ala874Thr]	c.[2620G>A]	14 y	ND	Y^d^	N	N	N	N	N	N	Y	[137]
XP12PF	p.[Arg138Phefs*33]	c.[409_410insTT]			1 y	ND	Y	N	ND	N	ND	Y	ND	ND	[138]
XP13PF^c^	p.[Arg138Phefs*33]	c.[409_410insTT]			1 y	ND	Y	N	ND	N	ND	N	ND	ND	[138]
XP101BR	p.[Ile290Asn]	c.[869T>A]			68 y	ND	Y	N	N	Y (50 y)	N	Y (50 y)	Y (50 y)	Y	[139]
XP119BR	p.[Glu585*]	c.[1753G>T]	p.[Ala818Val]	c.[2453C>T]	38 y	ND	Y	Y (20 y)	N	Y (> 30 y)	N	Y	N	ND	[139]
XP120BR^c^	p.[Glu585*]	c.[1753G>T]	p.[Ala818Val]	c.[2453C>T]	36 y	ND	Y	N	N	Y (> 30 y)	N	Y	N	ND	[139]
XP118BR	p.[Leu615Trpfs*17]	c.[1842delT]	p.[Ala795Thr]	c.[2383G>A]	10 y	ND	Y	N	ND	N	ND	Y^d^	ND	ND	[139]
XP174-1	p.[Leu778Pro]	c.[2333T>C]			22 y	ND	Y	N	N	Y^d^	N	N	Y	ND	[140]
XP174-2^c^	p.[Leu778Pro]	c.[2333T>C]			17 y	ND	Y	N	N	Y^d^	N	N	N	ND	[140]
XP29MA	p.[Leu778Pro]	c.[2333T>C]^e^			14 y	ND	Y	N	N	N	N	Y	N	Y	[141]
XP30MA^c^	p.[Leu778Pro]	c.[2333T>C]^e^			19 y	ND	Y	N	N	N	N	Y	N	Y	[141]
XP124LO	p.[Ala792Val]	c.[2375C>T]	p.[Glu960*]	c.[2878G>T]	14 y	ND	Y	N	N	N	N	N	N	Y	[142]
XP125LO^c^	p.[Ala792Val]	c.[2375C>T]	p.[Glu960*]	c.[2878G>T]	12 y	ND	Y	N	N	N	N	N	N	Y	[142]
XP34BR	p.[Ala818Val]	c.[2453C>T]	p.[Thr863Argfs*17]	c.[2586_2587delTA]	20 y	ND	Y	N	ND	Y	ND	Y	ND	Y	[139]
Patient case IV-1	p.Ala818Val	c.[2453C>T]			ND (adult)	ND	Y	N	N	N	N	N	N	ND	[143]
Patient case IV-2^c^	p.Ala818Val	c.[2453C>T]			ND (adult)	ND	Y	N	N	N	N	N	N	ND	[143]
XP31KO	p.[?]	c.[?]	p.[?]	c.[?]	37 y	ND	Y	Y (37 y)	N	N	N	N	N	Y	[144]
XP52HM	p.[?]	c.[?]	p.[?]	c.[?]	60 y	ND	Y	Y (54 y)	ND	N	ND	ND	ND	Y	[145]

Y, yes; N, no; y, year; ND, not determined or not described

^a^Putative phenotype based on described clinical data and disease progression

^b^Mutation or AA alteration is homozygous if second allele is not indicated

^c^Sibling of previous listed patient

^d^Mild symptoms

^e^Molecular analysis Dept. of Molecular Genetics, Erasmus MC

**Table 2:** XPCS complex patients with XPG/ERCC5 mutations

	Allele 1		Allele 2^b^				Clinical features individual								
XPCS Patient ID^a^	AA change	Genomic mutation	AA change	Genomic mutation	Age	Age of death	Photosensitivity	Skin Cancer	Developmental delay	Mental retardation	Skeletal abnormalities	ophthalmologic abnormalities	psychomotor impairment	Cellular DNA Repair defect	Reference
XP20BE	p.[Glu11*]	c.[31G>T]	p.[?]	c.[?]	6 y	6.1 y	Y	N	Y	Y	ND	Y	Y	Y	[64, 137, 146–148]
XP82DC	p.[Gln16*]	c.[46C>T]	p.[Val869Glyfs*11]	c.[2606_2607delTG]	3 y	5.8 y	Y	N	Y	Y	ND	ND	ND	Y	[137]
XP55BR	p.[Gln16_Leu88del, Val89Argfs*2]	c.[264 + 1delG]			15 y	ND	Y	ND	ND	Y	ND	Y	Y	Y	[139]
XP56BR^c^	p.[Gln16_Leu88del, Val89Argfs*2]	c.[264 + 1delG]			18 y	ND	N	ND	ND	Y	ND	Y	Y	Y	[139]
XP172MA	p.[Val17_Gln176del, Val29_Gln176del]	c.[?]			5 y	ND	Y	N	ND	Y	ND	ND	ND	Y	[149]
Patient case 5	p.[Gln37*]	c.[109C>T]	p.[Leu392*]	c.[1173dupT]	3 y	ND	Y	N	Y	Y	ND	ND	ND	ND	[150]
XP104BR	p.[His46Metfs*5]	c.[136delC]			6 y	ND	Y	N	Y	Y	ND	Y	ND	Y	[139]
Patient case	p.[Thr57Thrfs*28]	c.[170_171insTT]			6 mo	6.5 mo	ND	N	Y	Y	ND	Y	Y	ND	[151]
Patient case	p.[R69*]	c.[205C>T]			ND	ND	ND	ND	Y	ND	ND	ND	Y	ND	[152]
Patient case 10239	p.[R69*]	c.[205C>T]			321 d	356 d	ND	ND	ND	ND	ND	ND	ND	ND	[153]
XPCS4RO	p.[Pro72His]	c.[215C>A]	p.[Gln176*]	c.[526C>T]	11 mo	11 mo	Y	Y^d^	Y	Y	ND	ND	Y	Y	[154]
XP40GO	p.[Gln150*]	c.[448C>T]	p.[Leu778Pro]	c.[2333T>C]	ND	ND	ND	ND	ND	ND	ND	ND	ND	Y	[155]
26PO	p.[Gln176*]	c.[526C>T]	p.[Lys754Asnfs*46]	c.[2258_2258del]	15 m	2 y	Y	N	Y	Y	N	Y	Y	ND	[156]
XPCS1BD	p.[Glu225Serfs*19, Glu225_Gln231del]	c.[673-1G>A]			28 y	ND	ND	ND	ND	ND	ND	ND	ND	Y	[64]
XPCS2LV^e^	p.[Arg263*]	c.[787C>T]	p.[Ser659Valfs*2]	c.[1975delA]	11 mo	20 mo	Y	N	Y	Y	Y	Y	Y	Y	[157, 158]
CS33PV	p.[Gly294Alafs*10]	c.[881-26G>A]	p.[Leu918Ilefs*12]	c.[2752insA]	8 mo	12 mo	N	N	Y	Y	ND	Y	Y	Y	[159]
XP96TA	p.[Leu308Serfs*12]	c.[922_923delTC]			11 mo	6 y	ND	N	Y	ND	ND	ND	ND	Y	[137]
XP2BI	p.[Arg372Thrfs*6]	c.[1114_1117delAGGA]	p.[Leu858Pro]	c.[2573T>C]	17 y	ND	Y	N	N	Y (> 11 y)	N	N	Y (> 11 y)	Y	[157, 160, 161]
XP3BR	p.[Asp499Ilefs*24]	c.[1494delA]	p.[Lys917fs*65, Lys917fs962fs*1186]	c.[2751delA]	4.5 y	6 y	Y	N	Y	Y	ND	ND	Y	Y	[64, 160, 162]
XPCS1LV^f^	p.[Ser659Valfs*2]	c.[1975delA]	p.[?]	c.[?]	9 mo	6.5 y	Y	N	Y	Y	Y	Y	Y	Y	[157, 158]
XP72MA	p.[Glu727*]	c.[2179G>T]	p.[Trp814Ser]	c.[2441G>C]	7 y	ND	Y	N	ND	Y	ND	ND	Y	Y	[155]
Patient case 16	p.[Glu734_Thr1186 delins2*]	c.[2200-10C>G]			4.3 y	ND	Y	N	Y	Y	N	Y	Y	Y	[163]
Patient case 1	p.[Asp798Tyr]	c.[2392G>T]			3 y	6.5 y	Y	N	Y	Y	ND	Y	Y	ND	[164]
Patient case 2^c^	p.[Asp798Tyr]	c.[2392G>T]			5 y	ND	Y	Y	Y	Y	ND	Y	Y	ND	[164]
Patient case 3	p.[Asp798Tyr]	c.[2392G>T]			4 y	5 y	Y	N	Y	Y	ND	Y	Y	ND	[164]
XP165MA	p.[Gly805Arg]	c.[2413G>A]			2 y	2 y	Y	N	Y	Y	ND	ND	Y	Y	[155]
XPCS1RO^g^	p.[Gly926Alafs*56]	c.[2775delT]			7 mo	7 mo	Y	N	Y	ND	Y	Y	Y	Y	[165]

Y, yes; N, no; y, year; mo, month; d, day;  ND, not determined or not described

^a^Putative phenotype based on described clinical data and disease progression

^b^Mutation or AA alteration is homozygous if second allele is not indicated

^c^Sibling of previous listed patient

^d^No biopsy was taken to confirm skin cancer

^e^Alternative name is Patient BT

^f^Alternative name is Patient NF

^g^Alternative name is 94RD27

**Table 3:** COFS patients with XPG/ERCC5 mutations

COFS patient ID^a^	Allele 1		Allele 2^b^		Age	Age of death	Clinical features individual								
	AA change	Genomic mutation	AA change	Genomic mutation			Photosensitivity	Skin Cancer	Developmental delay	Mental retardation	Skeletal abnormalities	ophthalmologic abnormalities	psychomotor impairment	Cellular DNA Repair defect	Reference
Fetus case 1	p.[Leu345*]	c.[1034T>A]	p.[Gln680*]	c.[2038C>T]	27 WG	TOP (28 WG)	ND	ND	Y	ND	Y	Y	ND	Y	[166–168]
Fetus case 2^c^	p.[Leu345*]	c.[1034T>A]	p.[Gln680*]	c.[2038C>T]	12 WG	TOP (15 WG)	ND	ND	Y	ND	Y	Y	ND	Y	[166–168]
Fetus case 7	p.[Arg366*]	c.[1096C>T]^d^			2 d	2 d	ND	ND	Y	ND	Y	ND	ND	ND	[169]
Fetus case	p.[Ser448*]	c.[343C>G]			< 4 w	< 4 w	ND	ND	Y	ND	Y	ND	ND	ND	[170]
Fetus case 17-1	p.[Gln545*]	c.[1633C>T]			PB (26–29 WG)	~ 2 h	ND	ND	Y	ND	Y	ND	ND	ND	[171]
Fetus case 17-2	p.[Gln545*]	c.[1633C>T]			PB (26–29 WG)	~ 2 h	ND	ND	Y	ND	Y	ND	ND	ND	[171]
Fetus case 17-3	p.[Gln545*]	c.[1633C>T]			PB (26–29 WG)	~ 2 h	ND	ND	Y	ND	Y	ND	ND	ND	[171]
Fetus case 3	p.[Glu716Glyfs*3]	c.[2144dup]			27 WG	TOP (28 WG)	ND	ND	Y	ND	Y	Y	ND	ND	[166–168]
Fetus case UPN-0011	p.Asp809GlufsTer24	c.[2427delT]			ND	ND	ND	ND	ND	ND	Y	ND	ND	ND	[172]
Fetus case IV.1	p.[Leu923Thrfs*7]	c.[2766dupA]			24 WG	TOP (24 WG)	ND	ND	Y	ND	Y	Y	ND	ND	[167, 168]
Fetus case IV.2^c^	p.[Leu923Thrfs*7]	c.[2766dupA]			21 WG	TOP (21 WG)	ND	ND	Y	ND	Y	N	ND	ND	[167, 168]
Fetus case IV.6	p.[Leu923Thrfs*7]	c.[2766dupA]			21 WG + 5d	TOP (22 WG)	ND	ND	Y	ND	Y	Y	ND	ND	[167, 168]
Fetus case IV.11	p.[Leu923Thrfs*7]	c.[2766dupA]			20 WG	TOP (30 WG)	ND	ND	Y	ND	Y	ND	ND	ND	[167, 168]
Fetus case IV.12^c^	p.[Leu923Thrfs*7]	c.[2766dupA]			22 WG	TOP (22 WG)	ND	ND	Y	ND	Y	ND	ND	ND	[167, 168]
Fetus case 11	p.(Gly926Alafs*56)	c.[2775del]	p.[Val914Serfs*16]	c.[2739dup]	36 WG	TOP (36 WG)	ND	ND	Y	ND	Y	ND	ND	ND	[168]
Fetus case 12	p.(Gly926Alafs*56)	c.[2775del]	p.[Val914Serfs*16]	c.[2739dup]	28 WG	1 d	ND	ND	Y	ND	Y	ND	ND	ND	[168]

Y, yes; N, no; y, year; d, day; w, week; WG, week of gestation; ND, not determined or not described; TOP, termination of pregnancy; PB, premature birth

^a^Putative phenotype based on described clinical data and disease progression

^b^Mutation or AA alteration is homozygous if second allele is not indicated

^c^Sibling of previous listed fetus

^d^Allele 2 was maternally inherited by uniparental isodisomy of distal 13q

Like humans, model organisms such as *S. cerevisiae* and *C. elegans* with XPG deficiency show mild to severe growth and developmental defects [119, 120]. In addition, several XPG mouse models have been generated that recapitulate disease phenotypes. Strikingly, mice with point mutations inactivating XPG endonuclease activity or lacking the last 183 amino acids were UV sensitive, but otherwise normal, whereas mice lacking XPG completely or its last 360 amino acids (including half of the I domain) showed progressive growth retardation upon birth and died prematurely [121–123]. In line with this, conditional XPG knockout mouse models also showed shortened lifespan and many growth deficiencies including progressive progeroid features and neurodegeneration, whose severity strikingly depends on the genetic background of the mice [124].

It is still debated why truncations and certain mutations in XPG cause more severe phenotypes and in humans lead to progeroid CS features in addition to XP. Typically, CS is associated with hereditary defects in TC-NER proteins such as CSA and CSB, suggesting that XPG–XPCS mutations may reduce TC-NER besides GG-NER. However, mutations in the TC-NER protein UVSSA and some mutations in CSA and CSB do not cause CS, but the rather mild photosensitive disorder UV sensitive syndrome, indicating that mere TC-NER deficiency is not the underlying cause for the additional severe symptoms. Possibly, a function of XPG outside NER plays a role in disease development. Human patients and mouse models expressing intact but nuclease-inactive XPG do not typically show severe CS-like features, suggesting that it is not the loss of enzymatic activity but the loss of non-enzymatic and/or structural functions of XPG that is linked to a more severe phenotype. Because of this, multiple hypotheses have been put forward to explain the pathogenesis of CS involving non-NER functions for XPG and other NER proteins. For instance, because XPG strongly interacts with and stabilizes TFIIH, a feature that is disrupted by a CS-causative mutation, it was suggested that gene expression problems may give rise to some of the more severe CS symptoms [51]. Furthermore, possibly truncating XPG mutations may specifically impair non-catalytic roles of XPG such as in oxidative DNA damage repair [101, 102] or in homologous recombination and/or replication fork protection [116, 117], giving rise to more severe developmental defects as observed in CS. Oxidative DNA damage repair may be particularly relevant to neurological CS features because oxidative lesions occur endogenously in cells like neurons that are not exposed to the environment and because some types of oxidative lesions are repaired by TC-NER [125]. Notably, like XPG, the TC-NER factors CSA and CSB have also been implicated in repair of oxidative damage via BER. *CS-A* and *CS-B* patient cells are hypersensitive to oxidative DNA damage and show accumulation of oxidative lesions [94, 126–128]. Also, CSB has been reported to interact with multiple BER factors, such as PARP1 [129] and APE1 [130] and to promote recruitment of XRCC1 to sites of oxidative DNA damage [95]. It has been suggested that transcription blockage by accumulation of oxidative damage might trigger neuronal cell death or senescence [125, 131]. Therefore, it is possible that specific CSA, CSB or XPG mutations result in deficient removal of oxidative lesions, leading to blocking of transcription and, consequently, triggering of CS symptoms. Moreover, also the emerging role of XPG in R-loop processing may be relevant to some aspects of the CS pathology, as in the absence of XPG R-loop levels will increase that may cause genome instability [104, 108, 111]. This may be even exacerbated due to the absence of functional TC-NER, as transcription-blocking DNA lesions lead to spliceosome displacement which causes the mRNA to hybridize with template ssDNA and form R-loops [106]. Moreover, these R-loops were shown to trigger noncanonical ATM activation leading to alternative spliced transcripts and differences in gene expression that might adversely affect cell function. Despite these possibilities, we favor an explanation that takes into account the fact that only mutations in TC-NER genes, albeit not all, cause CS features, which strongly points to a pathogenic connection with the TC-NER process itself. We noticed previously that in XPF- and XPG-deficient XPCS complex cells the core NER machinery is continuously targeted to DNA damage due to the absence of repair [132]. Therefore, we proposed that additional CS features are caused by the inability to remove stalled NER intermediates, either involving Pol II or TFIIH, which would prevent repair of the lesion by other means and could interfere with transcription and replication [15]. More research is needed to prove the value of this and other hypotheses, focusing on the pathogenic impact of different disease-causing mutations in XPG on the exact molecular buildup of the TC-NER machinery and on TC-NER efficiency with regard to different types of endogenously occurring DNA damage.

In summary, although XPG was originally identified as a major endonuclease in NER, it has now become clear that the protein has important functions outside NER as well. Future research should be aimed at better understanding how the activity of XPG in these different pathways is regulated. In particular, it is not yet exactly clear whether XPG is recruited to the NER machinery separately or as part of TFIIH, whether it forms a dimer and when and how it dissociates after incision. Also, how its recruitment to and stable association with transcription sites, other types of DNA damage and repair pathways and R-loops are regulated is not known. Finally, a systematic comparison of XPG disease mutations and their impact on XPG functions and associated phenotypes in isogenic cellular or animal models may be helpful to elucidate how exactly its deficiency leads to certain disease symptoms.

### Supplementary Information

Below is the link to the electronic supplementary material.

Supplementary file1 (XLSX 24 KB)

## Data Availability

Not applicable.
